# Unification Still Nearer

**Published:** 1904-02

**Authors:** A. Jacobi, George Ryerson Fowler, E. Eliot Harris


**A Monthly Review of Medicine and Surgery.**

*EDITOR:*

WILLIAM WARREN POTTER, M. D.

All communications, whether of a literary or business nature, books for review and exchanges should be addressed to the editor: 284 FRANKLIN STREET, BUFFALO, N. Y.

VOL. XLIII.—LIX. FEBRUARY, 1904. No. 7

### Unification Still Nearer.

IN the issue of this journal for November, 1903, we printed an editorial article, giving a summary of the various steps taken during the past few years by the Medical Society of the State of New York, in the attempt to secure a unification of the profession through consolidation of the two leading state medical organisations,—the society and the association. The article referred to closed with the prediction that unification of the medical profession in the Empire State would be an accomplished fact by the time the Medical Society of the State of New York held its annual meeting in 1904. Some of the friends of the JOURNAL thought at the time this was an extravagant statement, because it would be a physical impossibility to bring this about so soon. We may explain that we did not intend to carry the idea that all the statutory requirements would be met and that the two bodies would be so soon amalgamated and operating under one head, with all the details of the consolidation working as a unit. What we did believe, and this we intended to convey, was that all would be arranged as far as committees could determine, that the plan for coalition would be reported to the society at the meeting referred to, and that the society in annual session assembled would pass upon the treaty.

If, then, this prophecy has not proved quite true, it has come very close to truth, for, according to the published reports in the weekly journals of January 16, 1904, a permission bill has been agreed upon. It was introduced in the senate, January 13, and at this writing (January 21) we are informed the bill has passed that body.[1] It is now in the assembly and will, no doubt, be dealt with by that house at an early day. Furthermore, a protocol

1. It has since passed the assembly and will become a law before the JOURNAL reaches the mails.

has been agreed upon by the commissioners to be submitted to the respective bodies, which it is believed will receive unanimous indorsement.

In order to present a historical record of the subject, we print the protocol and bill as an addendum to this article, both of which, probably, will become a part of the consolidation measures before this JOURNAL reaches our readers. We also print elsewhere an address to the medical profession of New York, by Dr. J. N. McCormack, of Kentucky, on the subject of medical organisation. Dr. McCormack is charged by the American Medical Association with the duty of promoting organisation where it does not exist, and of perfecting it wherever it is imperfect or inadequate. He makes an essential point when he declares the county society to be the unit whence all authority is derived. This, however, is not new in New York, where the method has prevailed for nearly a century.

As one of the earlier advocates of coalition, the editor of the JOURNAL feels a supreme satisfaction in the contemplation that a solution of this difficult question is so near at hand. It has presented many vexations features and has subjected men to criticism and misjudgment by their confrères. Upon the general question of union there can be little difference of opinion; upon the details, however, it is but natural that men should differ. Humanity is so constituted that all cannot view the same side of the shield at one and the same time, or in the same light. These differences, however, should not engender animosities or provoke discourtesies. The well formed opinion of every interested member is entitled to courteous consideration; every member is entitled to offer his opinion for what it is worth and, furthermore, such opinion is entitled to receive respectful consideration from those whose duty it may be to listen to him.

Over and above all, however, naturally will be considered the result attained. The bickerings, heartburnings, trials and physical strain will all disappear under the soothing influence of a reunited profession. In one solid phalanx it must now move forward, seeking to create the greatest scientific advancement the world has ever known; and in this laudable purpose no prouder part will be taken by the profession of medicine anywhere than in the Empire State.

---

### PLAN FOR AMALGAMATION.

New York, January 12, 1904.

The subcommittee of the joint committee on conference presents the following plan of consolidation of the Medical Society of the State of New York and the New York State Medical

Association for the information of the medical profession of this state.

The plan is the result of much labor, legal investigation, and careful thought and attention on the part of the committee; and the organisation recommended for the consolidated corporation is one which is now in successful operation in thirty-two states of the United States. It is based upon the form of government of the United States, and care has been taken not to interfere in any way with the autonomy of county organisations, or with their property or other vested rights.

We respectfully urge the members of the profession to promote by all proper means the passage by the legislature of the proposed act prepared by counsel for the committee, authorising the consolidation of the Medical Society of the State of New York and the New York State Medical Association. The proposed act will be introduced in the assembly or senate in due course, and, if passed, will authorise the consolidation upon terms to be agreed upon by the two corporations. The consolidated corporation will be known as the Medical Society of the State of New York.

Before the agreement for consolidation under the proposed act will become operative or effective for any purpose, it must be authorised or ratified by the vote of each corporation at an annual meeting, or else at a special meeting of each corporation called to vote upon the agreement, and it must also be approved by the county medical societies in affiliation with the Medical Society of the State of New York.

The agreement to be recommended by the committee for adoption in accordance with the terms of the act will contain provisions for giving effect to the following stipulations:

1. All assets and liabilities of the New York State Medical Association shall be transferred to and vested in and assumed by the Medical Society of the State of New York at the time of consolidation.

2. Expert accountants shall be employed to ascertain the assets and liabilities of each corporation, and their reports shall be submitted with the agreement for consolidation.

3. All members of the New York State Medical Association in good standing at the time of the consolidation shall be admitted to membership in the Medical Society of the State of New York.

4. All members in good standing of the county medical societies now in affiliation with the Medical Society of the State of New York shall be admitted to membership in the Medical Society of the State of New York.

5. All members in good standing of the New York State Medical Association shall be admitted to membership in the county medical societies for the counties in which they respectively reside.

6. All members in good standing of the Medical Society of the State of New York shall be admitted to membership in the county medical societies for the counties in which they respectively reside.

7. The plan of organization, constitution and by-laws of the New York State Medical Association, with such modifications as have been agreed upon by the committee, shall be adopted as the plan of organisation, constitution and by-laws of the Medical Society of the State of New York.

8. The following proposition shall be submitted by referendum to the vote of the members of the consolidated corporation, namely:

"The principles of medical ethics of the American Medical Association, being suggestive and advisory, shall be the guide of members in their relations to each other and to the public."

9. The Medical Society of the State of New York will petition the legislature for the passage of such further enabling act as may be necessary, if any, to carry the consolidation agreement into effect.

### AN ACT

### TO AUTHORISE THE CONSOLIDATION OF THE MEDICAL SOCIETY OF THE STATE OF NEW YORK AND THE NEW YORK STATE MEDICAL ASSOCIATION.

*The people of the state of New York, represented in senate and assembly, do enact as follows:*

Section 1. The Medical Society of the State of New York, incorporated by or pursuant to chapter one hundred and thirty-eight of the laws of eighteen hundred and six, entitled, "an act to incorporate medical societies for the purpose of regulating the practice of physic and surgery in this state," and continued by chapter ninety-four of the revised laws of eighteen hundred and thirteen, passed April tenth, eighteen hundred and thirteen, entitled, "an act to incorporate medical societies for the purpose of regulating the practice of physic and surgery in this state," and the New York State Medical Association, incorporated under chapter four hundred and fifty-two of the laws of nineteen hundred, may enter into an agreement for the consolidation of such corporations, setting forth the terms and conditions of the consolidation and the mode of carrying the same into effect.

Each corporation, party to the agreement, may petition the Supreme Court for an order consolidating the corporations, setting forth in such petition the agreement for consolidation and a statement of all its property and liabilities and the amount and sources of its annual income. Before the presentation of the petition to the court, the agreement must be approved by a majority of the vote lawfully cast at an annual meeting of each corporation, separately, or at a meeting of each corporation separately and specially called pursuant to its by-laws for that purpose, and a certificate of such approval, verified by the president and secretary of the meeting shall be annexed to the petition.

On presentation of the petition, the certificate of approval and the consolidation agreement, and on such notice to interested parties as the court may prescribe, and after hearing such interested parties as desire to be heard, the court may make an order for the consolidation of the corporations on such terms and conditions as it may prescribe.

When the order is made and duly entered, the corporations, parties to the agreement, shall be one corporation under the name "Medical Society of the State of New York," which shall not be deemed to be a new corporation, but to be a continuation of the Medical Society of the State of New York, incorporated in eighteen hundred and six. A certified copy of said order shall be filed in the office of the secretary of state. All the property belonging to the corporations so consolidated shall vest in the said Medical Society of the State of New York, which shall have all the powers, rights and privileges possessed by either corporation at or immediately prior to the consolidation, and which shall be subject to all of the liabilities of each corporation.

Sec. 2. This act shall take effect immediately.

Respectfully submitted,

A. Jacobi,
George Ryerson Fowler,
E. Eliot Harris.

